# Inner retinal oxygen delivery and metabolism in progressive stages of diabetic retinopathy

**DOI:** 10.1038/s41598-024-54701-w

**Published:** 2024-02-22

**Authors:** Mansour Rahimi, Farzana Hossain, Sophie Leahy, Norman P. Blair, Xuejuan Jiang, Mahnaz Shahidi

**Affiliations:** 1 Department of Ophthalmology, University of Southern California, Los Angeles, CA USA; 2 Department of Population and Public Health Sciences, University of Southern California, Los Angeles, CA USA; 3 Department of Ophthalmology and Visual Sciences, University of Illinois at Chicago, Chicago, IL USA

**Keywords:** Diagnostic markers, Diabetes complications

## Abstract

Previous studies have reported increased retinal venous oxygen saturation and decreased retinal blood flow and oxygen metabolism in non-proliferative diabetic retinopathy (NPDR). The current study aimed to determine alterations in both inner retinal oxygen delivery (DO_2_) and metabolism (MO_2_) in proliferative DR (PDR) as well as at stages of NPDR. A total of 123 subjects participated in the study and were categorized into five groups: non-diabetic control (N = 32), diabetic with no diabetic retinopathy (NDR, N = 34), mild NPDR (N = 31), moderate to severe NPDR (N = 17), or PDR (N = 9). Multi-modal imaging was performed to measure oxygen saturation and blood flow, which were used for derivation of DO_2_ and MO_2_. There were significant associations of groups with DO_2_ and MO_2_. DO_2_ was lower in PDR and not significantly different in NDR and NPDR stages as compared to the non-diabetic control group. MO_2_ was decreased in PDR and moderate to severe NPDR as compared to the control group, and not significantly reduced in NDR and mild NPDR. The findings demonstrate reductions in both DO_2_ and MO_2_ in PDR and MO_2_ in moderate to severe NPDR, suggesting their potential as biomarkers for monitoring progression and treatment of DR.

## Introduction

Diabetic retinopathy (DR) is a serious complication of diabetes and the leading cause of vision loss among working-age adults in developed countries^1^. Hypoxia is considered to play a significant role in the pathogenesis of DR. With progressive stages of DR, there is a thickening of the vascular basement membrane, loss of pericytes, endothelial apoptosis, and capillary dropout, ultimately leading to a hypoxic inner retina and upregulation of inflammatory mediators^2–4^. Retinal hypoxia also stimulates the production of vascular endothelial growth factors (VEGF), and anti-VEGF treatment has been shown to be effective in reducing diabetic macular edema and neovascularization, as well as delaying the progression of DR^5^.

For their function, retinal cells are dependent on a considerable amount of energy generated from availability of adequate oxygen to their mitochondria^6^. Therefore, hypoxia will adversely affect energy production, metabolism, and cell survival. Currently, no methods are available for measuring retinal tissue oxygen content in human subjects. Alternatively, essential information about the ability of cells to generate energy, maintain tissue viability and perform visual processing can be obtained by assessment of the rates of oxygen delivered by the retinal circulation (DO_2_) and oxygen extracted by the retinal tissue for metabolism (MO_2_).

DO_2_ is determined by total retinal blood flow (TRBF) and retinal arterial oxygen saturation (SO_**2**_). Reduced DO_2_ due to decreased TRBF may lead to retinal tissue hypoxia and impaired MO_2_, as indirectly assessed by retinal vascular SO_2_. Accordingly, alterations in both TRBF^7–13^ and SO_2_^14–19^ have been reported due to DR. Additionally, a trend of increased retinal venous SO_2_ with increasing severity of non-proliferative DR (NPDR) has also been demonstrated^15,18,20,21^. Furthermore, studies have reported a decrease in the arteriovenous oxygen content difference^22,23^, concomitant with reduced TRBF in mild to moderate NPDR^24,25^ and an increase in TRBF in no or mild NPDR^26,27^. However, DO_2_ changes at different stages of DR have not been reported.

Recent studies have reported alterations in MO_2_ due to DR. Specifically, a reduction in retinal oxygen extraction has been reported in no or mild DR in type 1 diabetics^26^, and MO_2_ was shown to be decreased in moderate to severe NPDR in type 2 diabetics^28^. We have developed a multimodal imaging technique for the assessment of DO_2_ and MO_2_^29,30^ and reported their variabilities in healthy and diabetic subjects with no DR or mild NPDR^31^. Recently, we reported alterations in these retinal oxygen metrics before and after treatment in a case report of proliferative DR (PDR)^32^. However, there is limited information about the combined assessments of DO_2_ and MO_2_ at all progressive stages of DR. The purpose of the current study was to test the hypothesis that DO_2_ and MO_2_ are altered in PDR as well as at stages of NPDR compared to non-diabetic healthy subjects.

## Materials and methods

### Subjects

The research study was approved by an Institutional Review Board of the University of Southern California. Prior to enrollment, the research study was explained to the participant, and informed consent was obtained in accordance with the Declaration of Helsinki. A total of 123 non-diabetic or type 2 diabetic subjects participated in the study, and one eye of each participant was evaluated. Based on comprehensive clinical examination by expert retinal specialists, the eyes were categorized into one of the five groups: non-diabetic control (N = 32), diabetes with no diabetic retinopathy (NDR, N = 34), mild NPDR (N = 31), moderate to severe NPDR (N = 17), and proliferative DR (PDR, N = 9). All PDR patients received panretinal photocoagulation (PRP) according to the clinical protocol. The time interval between treatment and imaging was 9.5 ± 7.0 months. Exclusion criteria were opacities of the ocular media, fixation instability that affected image quality, ocular conditions (such as retinal vascular occlusions, age-related macular degeneration, glaucoma), or refractive error greater than 6.00 diopter of myopia, or systemic diseases that are known to affect the retina (such as sickle cell disease). Non-diabetic controls less than 40 years of age were excluded. Intraocular pressure (IOP), mean arterial pressure (MAP), hematocrit (HCT), and HbA1c were measured. Ocular perfusion pressure (OPP) was determined using the formula 2⁄3 MAP – IOP.

### Image acquisition and analysis

Prior to imaging, participants’ pupils were dilated using 1% tropicamide and 2.5% phenylephrine. Multi-modal imaging was performed to measure TRBF and retinal vascular SO_2_, as previously described^29^. Volumetric images covering a 2 × 2-mm area centered on the central retinal vein were acquired using a commercially available Doppler OCT system (Avanti, Optovue Inc., Fremont, CA, USA). A previously established customized phase-resolved technique^29,30,33,34^ was used to determine blood velocity in individual vein branches on multiple *en face* planes. Using vein diameter measurements, TRBF was computed as the sum of flow in all veins^33,35^. TRBF measurements obtained from several images were averaged. Dual wavelength retinal oximetry was performed by acquiring images encompassing a 5 × 5-mm area centered on the optic disk using our custom-built slit lamp biomicroscope^36,37^. Retinal arterial and venous SO_**2**_ (SO_**2A**_ and SO_**2V**_) were determined for each blood vessel. To minimize SO_**2**_ measurement error, mean values in all arteries or veins of each eye were calculated and extreme SO_**2A**_ > 110% (95th percentile) were excluded. The variability of SO_2_ measurements was previously reported^37^. SO_**2A**_ and SO_**2V**_ values were converted to blood oxygen content of retinal arteries and veins (O_2A_ and O_2V_), using the oxygen-binding capacity of hemoglobin^38^ and hemoglobin concentration calculated from the measured HCT values. Mean values of O_**2A**_ and O_**2V**_ were determined by averaging measurements in all retinal arteries and veins, respectively. Retinal arteriovenous oxygen content difference (O_2AV_) was calculated as O_2A_ − O_2V_. DO_2_ and MO_2_ were calculated using the following equations: DO_2_ = TRBF × O_2A_; MO_2_ = TRBF × O_2AV_. The variabilities of DO_2_ and MO_2_ measurements were previously reported^31^.

### Statistical analysis

Retinal oxygen metrics (DO_2_, MO_2_) were the primary outcome variables evaluated. The distribution of outcome variables was examined for data normality using the Shapiro–Wilk test. Age was converted to a categorical variable. To determine differences in categorical variables (age, sex, and race) and continuous variables (HbA1c, MAP, IOP, and OPP) among the five groups (control, NDR, mild NPDR, moderate to severe NPDR, PDR), X^2^ tests and one-way Analysis of Variance (ANOVA) were used, respectively. General linear models (GLM) were generated to determine the association between groups (independent variables) and retinal oxygen metrics (dependent variables). Estimates of mean difference (β) with respect to the control group were determined. Pairwise comparisons between DR groups were performed using post hoc Least Significant Difference (LSD) tests. Data were analyzed using R version 4.3.1 (R Foundation for Statistical Computing). A significance level of p < 0.05 was established and all statistical tests were two-tailed.

## Results

### Demographic characteristics

The demographic and clinical characteristics of the subjects are presented in Table 1. Mean HbA1c differed among groups (p < 0.001), while age, sex, race, MAP, IOP, and OPP were not significantly different (p ≥ 0.09).

**Table 1:** Demographic characteristics of control and diabetic subjects at stages of DR (mean ± SD)

	Total (N = 123)		Control (N = 32)	NDR (N = 34)	NPDR, mild (N = 31)	NPDR, moderate to severe (N = 17)	PDR (N = 9)	P-value
	N	%						
Age (years)			56 ± 10	59 ± 13	61 ± 11	64 ± 10	60 ± 5	0.20
30–39	4	3.3	0	3	1	0	0	0.09
40–49	19	15.4	10	5	4	0	0	
50–59	41	33.3	10	8	11	7	5	
60–69	32	26	8	8	8	4	4	
≥ 70	27	22.0	4	10	7	6	0	
Sex								0.65
Male	52	42.3	12	13	13	10	4	
Female	71	57.7	20	21	18	7	5	
Race								0.28
Asian	17	13.8	8	3	3	3	0	
African American	16	13.0	7	3	5	0	1	
White/non-Hispanic	23	18.7	4	8	7	3	1	
Hispanic	67	54.5	13	20	16	11	7	
HbA1c (%)			5.5 ± 0.5	6.8 ± 0.8	7.5 ± 1.4	7.9 ± 1.5	8.2 ± 1.8	< 0.001
MAP (mmHg)			100 ± 13	99 ± 14	99 ± 15	102 ± 17	95 ± 9	0.85
IOP (mmHg)			15 ± 3	16 ± 4	14 ± 3	15 ± 4	16 ± 7	0.46
OPP (mmHg)			52 ± 9	51 ± 10	52 ± 10	54 ± 11	47 ± 10	0.55

*HbA1c* glycated hemoglobin, *IOP* intraocular pressure, *MAP* mean arterial pressure, *OPP* ocular perfusion pressure, *SD* standard deviation.

### Inner retinal oxygen delivery

Table 2 shows the mean and standard deviation (SD) of DO_2_ stratified by group. There was a significant association between the groups and DO_2_ (p = 0.02). Table 3 shows DO_2_ differences (β) relative to the control group. DO_2_ was significantly lower in the PDR group compared to the control group (p = 0.001) and also compared to NDR and mild NPDR groups (p = 0.02), but not different than moderate to severe NPDR (p = 0.08). There was no statistically significant reduction in DO_2_ in NDR or NPDR groups as compared to the control group (p ≥ 0.13). DO_2_ was lower in moderate to severe NPDR than the control group, but the difference did not reach statistical significance (p = 0.09).

**Table 2:** Mean and standard deviation (SD) of oxygen metrics stratified by group.

Group	DO_2_ (µLO_2_/min)	MO_2_ (µLO_2_/min)	N
Control	8.5 ± 3.0	3.1 ± 1.1	32
NDR	7.6 ± 2.1	2.8 ± 1.0	34
NPDR, *mild*	7.5 ± 3.0	2.7 ± 1.5	31
NPDR, *moderate to severe*	7.1 ± 2.9	2.3 ± 1.1	17
PDR	5.1 ± 1.7	1.8 ± 0.5	9

**Table 3:** Association of group and DO_2_ (β = estimate of mean difference, CL = 95% confidence limit).

Group	DO_2_ (µLO_2_/min)			
	β	P-value	Lower CL	Upper CL
Control	*ref*			
NDR	− 0.89	0.18	− 2.2	0.42
NPDR, *mild*	− 0.04	0.13	− 2.4	0.30
NPDR, *moderate to severe*	− 1.40	0.09	− 3.0	0.21
PDR	− 3.40	0.001	− 5.4	− 1.4

### Inner retinal oxygen metabolism

Table 2 presents the mean and SD of MO_2_ stratified by groups. There was a statistically significant association between the groups and MO_2_ (p = 0.02). Table 4 shows MO_2_ differences (β) relative to the control group. MO_2_ was significantly lower in the PDR group compared to the control group (p = 0.002), as well as compared to NDR and mild NPDR groups (p ≤ 0.03). MO_2_ was also lower in moderate to severe NPDR compared to the control group (p = 0.02). There was no statistically significant difference in MO_2_ in NDR and mild NPDR groups compared to the control group (p ≥ 0.16).

**Table 4:** Association of group and MO_2_ (β = estimate of mean difference, CL = 95% confidence limit).

Group	MO_2_ (µLO_2_/min)			
	β	P-value	Lower CL	Upper CL
Control	*ref*			
NDR	− 0.30	0.32	− 0.8	0.30
NPDR, *mild*	− 0.41	0.16	− 1.0	0.20
NPDR, *moderate to severe*	− 0.82	0.02	− 1.5	− 0.15
PDR	− 1.40	0.002	− 2.2	− 0.50

## Discussion

The findings of the current study confirmed our hypothesis that DO_2_ and MO_2_ were altered in patients with PDR compared to non-diabetic control subjects. Moreover, MO_2_ was also lower in moderate to severe NPDR than non-diabetic subjects. However, the results did not show a significant change in DO_2_ and MO_2_ in NDR or mild NPDR.

The findings of this study showed that DO_2_ was significantly reduced in patients with PDR compared to the non-diabetic control group, consistent with our previous case report study^32^. The eye employs a combination of vascular (myogenic) and metabolic autoregulation to ensure an adequate supply of oxygen to the retina^39,40^. While there are conflicting reports regarding the impact of diabetes on blood flow, impaired autoregulation and more severe vascular damage are recognized as the primary factors contributing to decreased blood flow in the PDR stage^41,42^. Despite the maximal compensatory response exhibited by autoregulatory vasodilation to augment blood flow, it remains inadequate in effectively handling the insult, thereby leading to alterations in blood flow and subsequently impacting DO_2_. Moreover, PDR patients in the current study had received PRP. This treatment, by the destruction of the outer retina cells, is likely a contributing factor to the observed reduction in DO_2_ (rate of oxygen delivered by the retinal circulation) due to increased oxygen diffusion from the choroid to the inner retina^43^. However, it is important to consider that the effects of PDR disease and treatment are not mutually exclusive and may be difficult to differentiate. Diabetes itself can initiate pathological changes in retinal oxygenation and metabolism. At the same time, PRP may exacerbate or modify these alterations in the context of PDR. There are two factors that determine DO_2_, namely, retinal blood flow and oxygen saturation. As discussed above, several studies have reported a reduction in retinal blood flow in treated PDR^32,44,45^, while other studies have demonstrated increased retinal arterial oxygen saturation^15–18,46^. Our findings of decreased DO_2_ and blood flow in PDR are indicative of the dominant determining role of blood flow. Nevertheless, further investigations are warranted to elucidate the interplay between altered blood flow and oxygen saturation in determining oxygen delivery, during the course of disease and following therapeutic interventions in PDR. In the current study, DO_2_ was not reduced in NDR or mild NPDR but tended towards reduction in moderate to severe NPDR. To our knowledge, changes in DO_2_ due to NPDR have not been previously reported. It is likely that the decrease in blood flow may be offset by an increase in oxygen content, resulting in a tendency toward maintenance of DO_2_. With additional investigations, the evaluation of DO_2_ may serve as a valuable approach in identifying potential contributions to the development and progression of DR.

The current study showed that MO_2_ was significantly lower in the PDR group compared to the non-diabetic control group. Prolonged hypoxia in PDR can lead to cellular degeneration and dysfunction, causing a decrease in the demand for oxygen as reflected in decreased MO_2_^16^. This finding agrees with our previously reported abnormal MO_2_ after treatments in a case of PDR^32^. Another factor that may affect MO_2_ is PRP treatment of PDR. Patients with PDR may experience decreased photoreceptor oxygen consumption, resulting in an increased supply of oxygen to inner retinal neurons via the choroidal circulation^43^. It has been previously reported that under hyperoxia, MO_2_ (the rate of oxygen extraction from the retinal circulation) can decrease in the inner retina if it is supplied by choroidal circulation^47,48^. This increase in oxygen availability from the choroidal circulation leads to a reduction in the rate at which oxygen is extracted from the retinal circulation since less retinal tissue is supplied by the retinal circulation. Our finding of lack of significant differences in MO_2_ between non-diabetic control, NDR, and mild NPDR groups is in agreement with a previous study^28^. Indeed, the ratios of MO_2_ in NDR and mild NPDR to control were similar between our study and the previous study; NDR (0.91 vs. 0.96) and mild NPDR (0.87 vs. 0.85). Moreover, consistent with the previous report^28^, our findings showed a decrease in MO_2_ in moderate to severe NPDR. The ratio of MO_2_ in moderate to severe NPDR to control in our study and the previous study was also similar: (0.74 vs. 0.79). In general, the lack of an objective and standard method for DR classification, differences in techniques employed for deriving MO_2_, study sample sizes, subjects’ demographics and clinical histories, and other confounding factors affect accurate comparison of results between studies. Future studies are needed to determine the factors that contributed to the observed MO_2_ reduction in moderate to severe NPDR. 

There are several limitations to this study. First, the study was conducted on a limited population at various stages of DR, and further investigation with a larger cohort study is necessary. Second, other potential factors that might impact MO_2_ and DO_2_, such as hypertension, hyperlipidemia, the duration of diabetes, the presence of diabetic macular edema, and history of treatment for DR, were not fully taken into consideration. Third, although pupillary dilation using phenylephrine may have affected retinal perfusion^49^, its consistent use across all subjects did not likely affect the findings. Fourth, hematocrit was measured from the peripheral vein and its value may be different in retinal vessels, though the difference likely contributed similarly to all data and had a minimal effect on group comparisons. Fifth, we did not account for differences in oxygen affinity of hemoglobin and glycosylated hemoglobin, which may have affected the accuracy of MO_2_ and DO_2_ measurements in diabetic subjects. However, since the mean difference in HbA1c between non-diabetic and diabetic groups was less than 2.7%, this factor likely minimally influenced the findings. Furthermore, the results of our statistical models did not change with inclusion of HbA1c as a covariate. Finally, longitudinal studies are required to define the observed changes further and differentiate the effects of disease progression and treatment.

In conclusion, the current study demonstrated reductions in DO_2_ in PDR and in MO_2_ in PDR and moderate to severe NPDR compared to non-diabetic subjects. Future longitudinal studies are needed to identify DO_2_ and MO_2_ as potential biomarkers for monitoring progression and treatment in DR.

## Data Availability

The data generated are available from the corresponding author upon reasonable request.
